# Investigating change across time in prevalence or association: the challenges of cross-study comparative research and possible solutions

**DOI:** 10.1007/s44155-022-00021-1

**Published:** 2022-10-27

**Authors:** David Bann, Liam Wright, Alice Goisis, Rebecca Hardy, William Johnson, Jane Maddock, Eoin McElroy, Vanessa Moulton, Praveetha Patalay, Shaun Scholes, Richard J. Silverwood, George B. Ploubidis, Dara O’Neill

**Affiliations:** 1 Centre for Longitudinal Studies, Social Research Institute, University College London, London, UK; 2 School of Sport, Exercise and Health Sciences, Loughborough University, Loughborough, UK; 3 Social Research Institute, University College London, London, UK; 4 MRC Unit for Lifelong Health and Ageing, University College London, London, UK; 5 School of Psychology, Ulster University, Coleraine, UK; 6 Department of Epidemiology and Public Health, University College London, London, UK

**Keywords:** Comparative research, Time trends, Cross-study analysis, Measurement, Missing data

## Abstract

Cross-study research initiatives to understand change across time are an increasingly prominent component of social and health sciences, yet they present considerable practical, analytical and conceptual challenges. First, we discuss the key challenges to comparative research as a basis for detecting societal change, as well as possible solutions. We focus on studies which investigate changes across time in outcome occurrence or the magnitude and/or direction of associations. We discuss the use and importance of such research, study inclusion, sources of bias and mitigation, and interpretation. Second, we propose a structured framework (a checklist) that is intended to provide guidance for future authors and reviewers. Third, we outline a new open-access teaching resource that offers detailed instruction and reusable analytical syntax to guide newcomers on techniques for conducting comparative analysis and data visualisation (in both R and Stata formats).

**Supplementary Information:**

The online version contains supplementary material available at 10.1007/s44155-022-00021-1.

## Background

Across the social and health sciences, addressing many key scientific or policy challenges requires an understanding of whether, and to what degree, a given population characteristic has changed over time. This may be a change in the occurrence, average level or distribution of an outcome of interest. For example, understanding whether obesity, depression, or other health outcomes have become more or less frequent across time can inform the need for public health interventions, and can provide clues to aetiology by contrasting such trends with changes in their purported determinants or confounding factors. Similarly, social science is concerned with investigating change across time in important demographic (e.g., parity, single parenthood) or socioeconomic (e.g., employment, social class) factors. Further important issues in multiple disciplines require understanding change in the magnitude or direction of associations—for example, changes in social mobility across multiple generations [1] or change across time in health inequalities [2, 3].

Comparative or cross-study research can effectively address such questions by leveraging existing data sources (typically observational) that cover different time periods. However, while such initiatives are increasingly prominent components of social and health sciences [4, 5], they are notoriously challenging to conduct—involving the collation and analysis of data from distinct and potentially heterogenous sources, with a need to ensure that collation is valid, sources of bias are avoided/minimised, and inferences are drawn appropriately. Differences in decisions around data selection (e.g., definition of the analytical sample), processing and analysis can qualitatively alter the conclusions drawn (e.g., in the case of social mobility [6] or health inequality [7] trends across time). Despite the importance of tackling the methodological and conceptual challenges in pursuing such comparative research, examining single studies (understandably) remains the focus of existing methodological training, and to our knowledge there is a lack of dedicated resources to help in the transition to analysing and comparing estimates from multiple studies.

In the main body of this paper, we expand on this research approach, discussing in more detail the methodological issues commonly faced in such research and the different considerations required in adequately and appropriately surmounting such challenges. We build on previous papers and books which have offered insight on topics of relevance—including retrospective data harmonisation [8], pooled analysis of multiple studies [9–12], and investigation of changes in health inequality [2, 13]. The myriad of possible research questions in such comparative research means that we do not propose authoritative rules on best practice, but rather we offer suggestions on issues to be considered and possible options to address them. The discussion informs and explains a proposed checklist to help scaffold and guide effective cross-study research, and this could inform the development of best-practice in future (see Table 1). The checklist was developed using the STROBE guidelines for observational studies [14] as a template and through a series of knowledge exchange events amongst the study authors, and iteratively developed via subsequent discussion on draft versions.

**Table 1:** Checklist for studies which investigate differences in prevalence or associations across time

Domain Section	Recommendation
Rationale	Explain the scientific background and rationale for the comparative design; give (if any) prespecified hypotheses with supporting evidence where available
	Provide explanation of the basis for study selection/inclusion
Methods	
Study design	Present key elements of each study used, noting key similarities/differences in: (a) Target population (b) Sample recruitment (c) Exposure/outcome measurement (the measures validity and measurement protocols) (d) Covariate availability and specification For longitudinal analyses, provide any relevant detail on cross-study alignment in respondent age at assessment (where relevant) and interval lengths between data collections Provide sufficient and accurate citation of source data
Statistical methods	Give the rationale for statistical tests undertaken—where either simple or complex models are used
	Consider testing associations in both absolute and relative magnitudes, since conclusions may differ when only one is examined
	Note how cohort differences in association will be compared (e.g., informally by comparing effect estimates, and/or formally via meta-analysis/inclusion of cohort* exposure interaction terms)
	Identify, implement and document an appropriate missing data handling strategy
Estimation	
Results	Provide effect size/s and appropriate indicators of precision (e.g., 95% CI); comment on the size of the cohort difference in association
	Where appropriate consider accounting for confounding variables (common causes of both exposure and outcomes); where included, provide unadjusted and confounder-adjusted effect estimates
	Consider sensitivity analyses to test the robustness of the associations observed; for instance, do conclusions differ when restricted to more comparable target populations (even at the expense of study power)
Inference	
Explanation of findings	Consider, using relevant supporting evidence, the potential explanation for cohort differences/similarities in the association observed: (a) Differences in causal effect of the exposure (b) Alternative explanations, for example differences in confounding/sample composition or measurement
Methodological considerations	Discuss the degree to which analyses are likely to be sufficiently powered to detect differences by cohort (e.g., note in the discussion or where credible a-priori rationale exists for differences in effect size)
	Include a balanced discussion of the strengths and limitations of the work undertaken e.g., whether the number of studies included and the timespan covered are sufficient
Implications	Rationalise the need for future research
	If appropriate give cautious implications for policy, based on the current study and other sources of evidence

In the proposed checklist and the wider discussion offered in this paper, we focus on the investigation of change across time in prevalence or association, but note that the considerations raised are similarly relevant to other forms of cross-study research (e.g., inter-country comparisons, see [15–18]). We discuss issues which are important for research which is descriptive in nature (i.e., one that aims to quantify some feature of a population) [19] and also for studies that seek to investigate a particular causal factor.

## Setting the scene

Newcomers to cross-study research may find the analytic complexities challenging, and as we set out in this paper, a wide range of methodological solutions may be employed. However, to illustrate the utility of this research approach and frame the discussion offered in this paper, we first present a new open-access teaching resource to demonstrate key steps that can be followed in pursuing cross-study research (accessible in an online format at https://ljwright.github.io/cross-cohort-tutorial/r_syntax.html and https://ljwright.github.io/cross-cohort-tutorial/stata_syntax.html). This new resource presents guided examples on core considerations and key methods for conducting cross-study analysis efficiently and effectively. It includes annotated re-usable code for the derivation of descriptive and inferential results across multiple cohorts/study sources (for both binary and continuous outcomes), and the visualisation of such results graphically. This is provided in both R and Stata formats, with guidance given on all supporting packages used. The scope and structure of the resource is explained in Box 1.

**Table:** 

Box 1. Teaching resource
The teaching resource comprises illustrative guidance on the following aspects of a comparative research workflow: • Descriptive statistics • Study-specific regressions • Meta-analysis • Pooled cohort regressions • Missing data • Modelling longitudinal data

### Which studies to include?

**Fig. 1:**
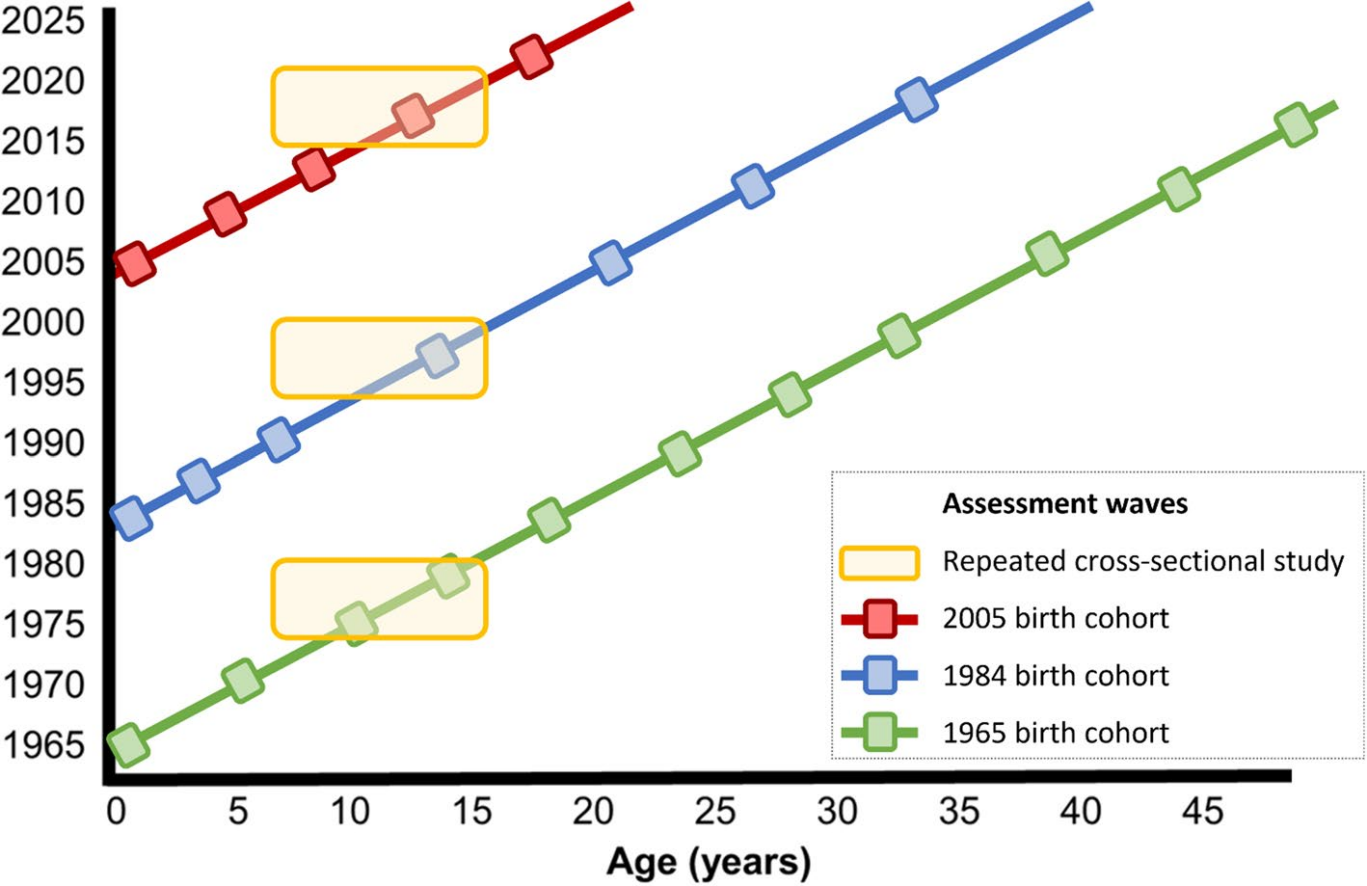
Depiction of different study designs to investigate change across time in prevalence or association: repeated cross-sectional studies and cohort studies in which comparisons can be made across childhood/adolescence using both sets of studies in 1975–1980, 1995–2000, and 2015–2020; or only using the cohort studies at other ages/time periods. Year(s) of data collection shown on the Y axis

Given the scope of longitudinally comparative research, multiple data sources (herein referred to as studies) covering different time periods are commonly required. A first consideration is which type of study to draw upon given the research question under investigation. Understanding whether prevalence or an association differs across time is addressable by a range of observational study designs in which results from two or more data-points are compared. For instance, such work could comprise the comparison of cohort studies or repeated cross-sectional studies (Fig. 1). Both enable investigation of change across time due to either the particular period investigated or cohort born into [20, 21]. Each has complementary strengths and limitations.

Cohort studies typically measure a comparatively large number of participants at specific ages, and their longitudinal design can be used to investigate age effects such as age-related changes in exposure or outcome, or indeed age-related changes in association. The temporal ordering of variables is additionally useful to account for confounding factors (i.e., common causes of exposure and outcome that are hypothesised to not lie on the causal pathway). In contrast, repeated cross-sectional studies typically sample a broader age span, thus improving the generalisability of findings beyond a specific birth cohort, albeit at the expense of power and potential representativeness of age-specific sub-groups. New rounds of sampling at each measurement occasion help to ensure the sample reflects recent demographic changes (e.g., recent migrants) which can be challenging to include in historically initiated cohorts. Repeated cross-sectional studies typically collect data at more regular intervals than do birth cohorts, and so can allow greater chronological precision, but lack the capacity to track the longitudinal sequencing of intra-individual change.

Cross-study research efforts typically focus on data based on the same study design. This has practical advantages in minimising between-study differences, yet may be a product (at least in part) of the specialisation of different research groups. For example, separate papers have investigated differences across time in obesity and its socioeconomic inequality in using repeated cross-sectional [22–24] or cohort [25, 26] study data. Similar examples are available from research into mental health, with researchers separately drawing on either repeated cross-sectional [27] or cohort [28] studies to investigate changes across time in levels of psychological distress/common mental disorders. Both study designs have merit; indeed, data from different study designs may be analysed together to leverage those respective strengths in addressing the same research question (Fig. 1 and see [29, 30]). Moreover, such work is facilitated by the availability of multiple existing cross-sectional and longitudinal studies that enable cross-study research, as are research and harmonisation resources. An illustrative list of these is provided in Table 2.

**Table 2:** Illustrative resources to aid investigation of change across time in prevalence or association

Study/resource type	Name of resource	Location, years	Website
Cross-sectional studies	European social survey, Europe	Europe, 2001	https://www.europeansocialsurvey.org/
	Health-oriented studies (NHANES and	USA, 1999	https://www.cdc.gov/nchs/nhanes/index.htm
	Health Survey for England)	England, 1994	http://healthsurvey.hscic.gov.uk
Longitudinal studies	SHARE	Europe, 2004	http://www.share-project.org/
	Birth cohort studies	UK, 1946	https://cls.ucl.ac.uk/cls-studies/
	Household panel studies (Understanding Society and PSID)	UK, 1991	https://www.understandingsociety.ac.uk
		USA, 1968	https://psidonline.isr.umich.edu/
Resources and harmonisation initiatives	CLOSER	UK	https://www.closer.ac.uk/
	Gateway to Global Aging	International	https://g2aging.org/
	Maelstrom	International	https://www.maelstrom-research.org/
	Cross-National Equivalent File	International, 1970	https://www.cnefdata.org/
	Multinational Time Use Study	International, 1960	https://www.timeuse.org/mtus
	Harmonized Learning Outcomes (HLO) database	International 2000	https://datacatalog.worldbank.org/search/dataset/0038001
	IPUMS	USA/worldwide, 1790	https://www.ipums.org/

This is a non-exhaustive list. There exists a multitude of social and health-oriented cross-sectional and longitudinal studies worldwide

For all study types, care should be taken in ensuring comparable target populations in the selection of studies to include. The target population may differ for example due to variation in sampling design. Many cohort studies are regional in nature (e.g., the Avon Longitudinal Study of Parents and Children [31]—based in South West England), and their use in cross-study research could therefore conflate regional differences with other comparisons of interest (e.g., those attributable to year of birth).

While a minimum of two studies are required for comparison, using three or more is generally preferable to correctly identify overall or generalised trends; comparisons of only two timepoints may be particularly susceptible to bias, since error in either estimate could then substantially distort the estimate of change [32]. The timespan investigated may also influence the conclusions drawn regarding trends across time in social or health phenomena. For example, a decline in community participation in the US has been repeatedly noted in recent decades [33, 34], yet recent work [35] found that it increased up to the 1960s and declined thereafter, offering new insight on the determinants of community participation and how such levels may be increased in future. Therefore, researchers should understand and specify the period of time they wish to study.

In summary, the selection of studies—in terms of type, number, and coverage/span—can be important and is thus worthy of deliberation. Choice should be primarily guided by knowledge of the specific topic being investigated and subsequently the data that is available to address the research question. Such choices are important in interpreting results since they will inform the generalisability of findings with respect to the target population(s), timespan, and inter-generational coverage (see Table 1).

### Exposure, covariate and outcome measurement

A second consideration is the measurement of key variables between the studies compared—specifically the outcome(s) and exposure(s), and potentially any covariates/confounders of cross-study relevance. Change across time in prevalence or association could reflect a true finding of real scientific and/or policy merit, or alternatively, be caused by methodological artefact (e.g., due to random or differential measurement error). Study differences in prevalence or association could feasibly be biased, or entirely confounded by, differences in measurement properties between studies [36]. For any difference in association to be inferred correctly, each key variable should be sufficiently comparable between studies.

Replicability of change across time in prevalence or association across different studies (including different study designs) can provide pragmatic insight on the pertinence of differences in measurement methods. For example, the finding that obesity prevalence has increased from the 1960s onwards is evident across multiple sources—repeated cross-sectional [37] and cohort studies [26]—stimulating a series of policy initiatives [38]. In this example, the prevalence difference across time is so large (e.g., worldwide, a 47% increase in overweight and obesity among children from 1980 to 2013 [37]) and consistently found that it is evidently unlikely to be attributable to differences in measurement across studies.

Over time, responses to the same survey questionnaire items may change. For instance, some [28, 39, 40] but not all [41] studies have suggested that common mental health problems has increased in recently born generations in the UK. However, so too has the awareness of mental health problems [42]—as may have the willingness to report such problems in surveys [43]. These in turn may influence the comparison of mental health prevalence levels across time, leading to inflated estimates of increase in more recent birth cohorts [44]. However, changes in reporting may not have affected the rank ordering of mental health difficulties in the population [45]. This would imply that comparisons of associations (where mental health is either the exposure or the outcome) would be valid across studies from different time points, even if the overall prevalence is not comparable. The same challenge in monitoring levels across time extend both to objectively assessed variables and to situations in which the method of measurement changes between studies (e.g., from Dinamap to Omron monitors in blood pressure measurement [29]). In each scenario, the likelihood of the associations being comparable should be appraised.

A related issue is that of panel conditioning; where the act of participation itself influences response in a future follow-up [46, 47]. This could potentially bias comparisons across time of longitudinal studies which differ in the number of follow-ups, thus further motivating the triangulation of evidence across different study designs (e.g., longitudinal and repeated cross-sectional studies). Multiple factors at the design stage may help to mitigate the likelihood of such bias (e.g., the length of time between longitudinal follow-ups, and careful design of question wording to avoid catalysing behaviour change); its occurrence and impact in terms of bias is likely to differ depending on the specific research question and questionnaire items investigated.

For the purposes of comparing associations, if the outcome is measured with three or more indicators (e.g. three distinct questions in a survey) and an underlying continuum is assumed, metric invariance, as it is known in the psychometric literature [48], can be tested with latent variable measurement models. Formal tests of metric invariance assess the assumption that the relative contribution of each measured indicator to the underlying construct is equal across groups (e.g., cohorts) or time (waves within cohorts). For instance, imagine three questions (measuring low mood, guilt, and decreased motivation) were used to assess a latent construct of depression in two distinct cohorts. If metric invariance is supported for this construct, this would suggest that ‘low mood’ was associated with, or weighted towards, depression to a similar degree across both cohorts. If metric invariance holds (as typically inferred on the basis of fit indices before and after equality constraints have been placed on measurement parameters (the ‘factor loadings’—loadings of the items on the latent construct) across groups) [49], then comparisons of associations (e.g., correlations, regression coefficients) across studies are unlikely to be biased by differences in outcome measurement across studies.

A more restrictive form of invariance, scalar invariance, can be tested for mean comparisons. Tests of scalar invariance assess the assumption that all of the between-group mean differences are captured by differences in the latent construct, as opposed to differences in measurement error [48]. Scalar invariance is tested by holding item intercepts/thresholds and factor loadings equal across groups. Non-invariance of an observed survey item indicates that between-group differences in that item are not driven solely by the underlying construct. For instance, non-invariance of a ‘decreased motivation’ item across two cohorts would indicate that mean differences in this item are not entirely due to differences in the levels of depression between the two groups.

These methods can, with assumptions, correct for differences in measurement error and therefore help enable cross-study research. For example, recent psychometric work has supported scalar invariance across a range of measures of mental health and cognitive ability in several British cohorts [45, 50, 51]. However, in practice, it is not uncommon for tests of measurement invariance [52], particularly the more stringent assumption of scalar invariance, to fail. This is more likely to occur when the number of groups (e.g., different studies and/or assessment waves) is large. As such, some methodologists suggest that tests of ‘exact’ measurement invariance are overly stringent, and propose alternative methods for when scalar invariance fails. In such instances, researchers could explore: (i) partial measurement invariance (wherein specific non-invariant parameters are freed across groups) [53], or (ii) novel approaches such as approximate measurement invariance (which require parameters to be approximately rather than exactly equal) [54]. Moreover, invariance testing is not applicable to single indicator observed outcomes; here, evidence from other studies should be used to inform the likelihood of bias, including the use of calibration studies [55–58].

Differences in random measurement error may also bias cross-study comparisons, and should therefore be considered in interpretation. If an exposure variable in one study has more random measurement error than its comparator, this would generally result in a weaker magnitude of association due to regression dilution bias [59]; if instead the outcome variable had more random measurement error then the effect size would be unchanged but the association would be less precisely estimated [59].

Differences in the availability or measurement of key covariates can also be important to consider to minimise bias of comparisons in prevalence and association across time—particularly where the same confounding structure exists in each study. Heterogeneity in the definition/operationalisation of such variables may be resolvable via retrospective harmonisation, where a valid basis for deriving equivalent scaling or categorisation is identified in each study. However, such harmonisation can lead to less informative scales being used—the lowest common denominator where variables overlap across studies. Where this is the case, the likely impact of such simplification on the comparison across time should be considered when interpreting results. In some instances, calibration methods including latent variable modelling may offer an appropriate statistical method for deriving equivalent measures (e.g., see [55, 60]) [61], but adherence to the required conditions needs testing and verification [62]. Where clinical cut-points are used, they may differ across time (e.g., reflecting changes in guidelines)—to avoid misclassification, the same cut-points should be used in the periods examined.

In summary, a key step in comparative evaluations of prevalence or association across time is therefore documentation, examination and quantification of between study heterogeneity in exposure, covariate and outcome measurement (see Table 1).

### Analysis

Assuming sufficiently comparable exposure, covariate and outcome variables are utilised, it is important to design and implement an appropriate analytical strategy. That is, one which facilitates comparisons between studies, minimises avoidable bias, has adequate power, and yields estimates which aid interpretation of the difference across time in prevalence or association.

The analytic approach should be sufficiently comparable across studies. That is, the same target quantity (i.e., estimand)—for instance, an average or local treatment effect—and comparable model specification (for instance, the same linear regression model form). This can be directly achieved where different data sets are pooled to enable concurrent analysis (sometimes termed integrative data analysis [12]), but also through coordinated [63] or ‘federated’ approaches where the implementation of analysis is devolved between study teams, avoiding the need for centralised data collation or access.

Differences in sampling between studies should be considered for each research question addressed. For instance, there are multiple national birth cohort studies in the UK, yet pertinent differences exist in their sampling—the 1946 cohort sampled singleton births of married women in mainland Britain [64]; restrictions not made in subsequent birth cohorts. Similarly, the Millennium Cohort Study sampled—indeed, oversampled—participants from Northern Ireland which were then followed-up [65]; the preceding national birth cohorts (initiated in 1946 [64], 1958 [66], and 1970 [67]) did not. These sources of between-study differences are in many cases potential sources of bias. However, in some cases the researcher may be explicitly interested in how the composition of the sample has changed over time and how, in turn, it might influence the change in prevalence or association under examination. For example, across time ethnic diversity has markedly increased in the UK and in many other high-income nations, and this may lead to differences in prevalence or associations amongst factors which differ by ethnicity in more recent sources of data.

To inform/control whether such target population differences influence the results of interest, studies can be restricted or weighted to comparable target populations in either main or sensitivity analyses. Differences between weighted and unweighted results may be expected where there is heterogeneity in prevalence or association by sub-group. For example, while obesity is strongly socioeconomically patterned in the UK [25] and in other high-income nations [68], the magnitude of association is seemingly larger in White compared with Black/minority ethnic groups [69, 70].

Differences in the patterns and/or magnitude of missing data may also bias cross-study comparisons. This is particularly pertinent given secular reductions in response rates to surveys in recent decades, evident in both cross-sectional [71] and cohort studies [25]. In cross-sectional studies this is manifested by failure to respond to the initial sampling invitation or to subsequent stages of the assessment process (e.g., participation at the interview but not health examination stage); or in cohort studies additionally by loss to follow-up in subsequent sweeps. Both sources of missing data (non-response and attrition) may be predicted by common factors—being in worse socioeconomic circumstances, and worse health for example. While avoiding such missing data is a primary goal (and a subject of survey methodology research [72, 73]), where it exists, analytical tools are available in both designs to use available information to correct to some extent for such missing data. A strength of cohort studies (relative to repeated cross-sectional studies) is the availability of earlier data sweeps which can be used to understand and subsequently redress missing data occurring in later sweeps. Due to the hierarchical nature of data collection for cross-sectional health examination surveys, information collected at the interview stage can be (and is) used to account for missing data at the nurse visit.

An immediate concern with missing data is loss of statistical power. While the sample sizes in each study do not need to be the same in order to make cross-study comparisons, smaller sample sizes in a given study lead to less precise estimates and lower statistical power. Another concern is potential bias in the estimate of change in prevalence or association and corresponding loss of sample representativeness—this depends on: (i) the extent of missing data; and (ii) the processes which led to such missingness occurring. All analytical options which either ignore or address missingness have assumptions, the plausibility of which is key to obtaining unbiased estimates [74, 75]. Conducting complete case analysis—that is, not explicitly accounting for missingness—may be appropriate where the amount of missingness is low, and may return unbiased (or less biased) [75, 76] results in some scenarios. However in many cross study comparisons, missingness may be substantial and affect the exposure, outcome and/or potential confounders, strengthening the case for using a principled approach to correct for missing data. Multiple studies have found that loss to follow-up in longitudinal studies is not entirely random—instead it is predicted by lower socioeconomic status, worse health, and lower levels of cognitive functioning, amongst other factors [77–81].

Assuming that missingness can be predicted sufficiently well by observed variables (the ‘Missing at Random’ assumption [79]), principled methods like multiple imputation, full information maximum likelihood (FIML) or inverse probability weighting should be employed to maintain power, restore sample representativeness across key variables and reduce bias [82]. Each of the methods has relative merits—for instance, multiple imputation enables additional auxiliary variables (those not included in the eventual analytical model) to be included to predict missingness; FIML allows their inclusion too under somewhat stronger assumptions, yet is typically more straightforward to specify in analytical syntax. Non-response weights are seemingly the default analytical approach for some data sources, as they are provided to users by the data owners. These are available in some but not all studies—and even within studies their coverage may not be consistent; to estimate change over time in prevalence or association, researchers could either manually create non-response weights or use alternative approaches such as multiple imputation or FIML. While non-response/attrition weights are relatively straightforward to include, their use relies on their external derivation—they are typically generic and thus may not necessarily remove pertinent biases in all research questions; further, they are generally less efficient than methods such as multiple imputation or FIML [83].

Where observed variables do not provide an unbiased prediction of missing values (i.e., where data are ‘Missing Not at Random’; MNAR), researchers can use methods such as pattern mixture modelling (PMM)[84] to estimate quantities of interest (means and associations) assuming specific missingness patterns. In PMM, researchers impute and then perturb missing values to reflect assumed MAR violations before using the perturbed data in final analytic models. Results which are robust to plausible—and more so, implausible—violations of MAR can be treated with more confidence. For examples of PMM in cross-study research, see [85, 86].

When imputing data in multiple studies, using study-specific imputation models is likely to be preferable to preserve differences between studies in means or covariances. We note that while the approach to missing data should ideally be comparable in each study, the specific variables used to handle missingness may differ in the studies compared. If different factors predict missingness between studies, then different variables would be needed to obtain unbiased estimates of study differences in association. If the ability to predict missingness between studies is due to differences in the availability of these predictors then bias may differ between studies.

Changes in associations over time can be examined on the relative (e.g., risk ratio, odds ratio) or absolute (e.g., risk difference) scales. We recommend that both are examined where possible as the choice can have profound effects when interpreting cross-study differences in associations (see accompanying teaching resource for illustrative code). This issue has been particularly paramount in the health inequalities literature [7], in which investigating the changing magnitudes of association between socioeconomic factors and health is a key aim despite substantial underlying changes to average population-level health (e.g., increased obesity prevalence [37] or reduced premature mortality rates [87]). Where the prevalence of the outcome differs across time, studies of different time periods can still find stability in odds ratios—but when analysed in absolute terms (e.g., risk differences), the magnitude of associations can systematically differ (see Supplementary Table 1 for a hypothetical worked example). For continuous outcomes, estimates are typically only presented on the absolute scale, yet changes over time can also be examined on the relative scale (e.g., using log-transformed outcomes, thus yielding percentage differences).

Another source of between study difference in association is changes over time in the distribution of the study sample across the key exposures of interest. For instance, researchers may wish to understand how associations between social class and health have changed across time; yet declining industrialisation in many high-income countries has led to a substantial reduction in the number of individuals employed in manual occupations [88, 89]. Thus, simple comparisons of ‘manual’ and ‘non-manual’ social classes likely compares vastly different fractions of the total populations of interest, and the characteristics of those in each class (e.g. gender, educational status, ethnicity, age) is likely to be different too. This may impair the comparison of change across time, even when the same categories are used in each study. The same issue is also a challenge with variables such as education, where attainment levels have markedly increased across time (e.g., in UK from 8 to 9 mean years of education in 1970 to 13 years by 2009 [90]) [91]. Therefore, differences in association between populations may be driven by differences in causal effects, but could also arise from changes in composition, if causal effects are heterogeneous and differences in composition mean that different sets of individuals are being “treated”. Whether this is considered a source of bias, or indeed of substantive research interest, depends on the research question. This potential for selection bias to influence cross-study differences in association [92] can be considered at the interpretation (see Sect. 2.4 below) as well as the analysis stage. In the health inequalities literature, this has been addressed by constructing exposure variables which are weighted to account for differences in the distribution of participants across categories of socioeconomic position (SEP) over time—for example, the relative index of inequality [93], in which exposures are converted into ridit scores—that is, each category in a dummy variable is assigned a value proportionate to its sample size from between 0 and 1. Such methods facilitate comparisons of association across the entire distribution of the exposure, assuming a linear relationship exists, yet do not fully rule out differences in composition (or selection) as causes of between-study differences in association [94]. Alternatively, regression or matching techniques may be used to identify and test comparable groups across populations [40]; in such analyses, internal comparability across studies is maximised at the possible expense of external generalisability to the target population.

Assuming that comparable exposures are identified in each study, cross-study differences in association can be compared informally and/or formally. Means of formal testing include estimating study-by-year interaction terms in a pooled dataset (see accompanying syntax). Historically however, this has typically been interpreted using only the resultant significance values which are not in themselves informative of the magnitude of any differences in association, and are frequently interpreted in binary terms [95] which is problematic given the large sample sizes required to detect interaction effects [96]. Instead, an indication of effect size (such as the coefficient of the study-by-time variable) and an indicator of its precision (e.g., confidence intervals) could be presented along with study-specific estimates of association. Interaction terms are tested in models pooling individual level data across multiple studies. This is challenging to do where studies differ in their sample design, such that sample weights or clustering differ. In such scenarios, weights can be constructed in all included studies (e.g., giving a weighting value of 1 in studies in which there was no oversampling [97]). Alternative strategies to formally test study differences in association, without the need for comparable sampling strategies, include the use of meta-analysis and meta-regression [98]—here, study-specific estimates of association are outputted and then subsequently compared (see accompanying syntax for example code).

In summary (see Table 1), consistency in analytic approach, including the treatment of missing data, can help reduce additional sources of bias in cross-study research. The choice of estimand for associations should be made with deliberation, and changes over time in the composition of specific correlates may warrant consideration. Researchers can pursue either informal or formal approaches for comparing such associations.

### Sources of different results between cohorts

**Fig. 2:**
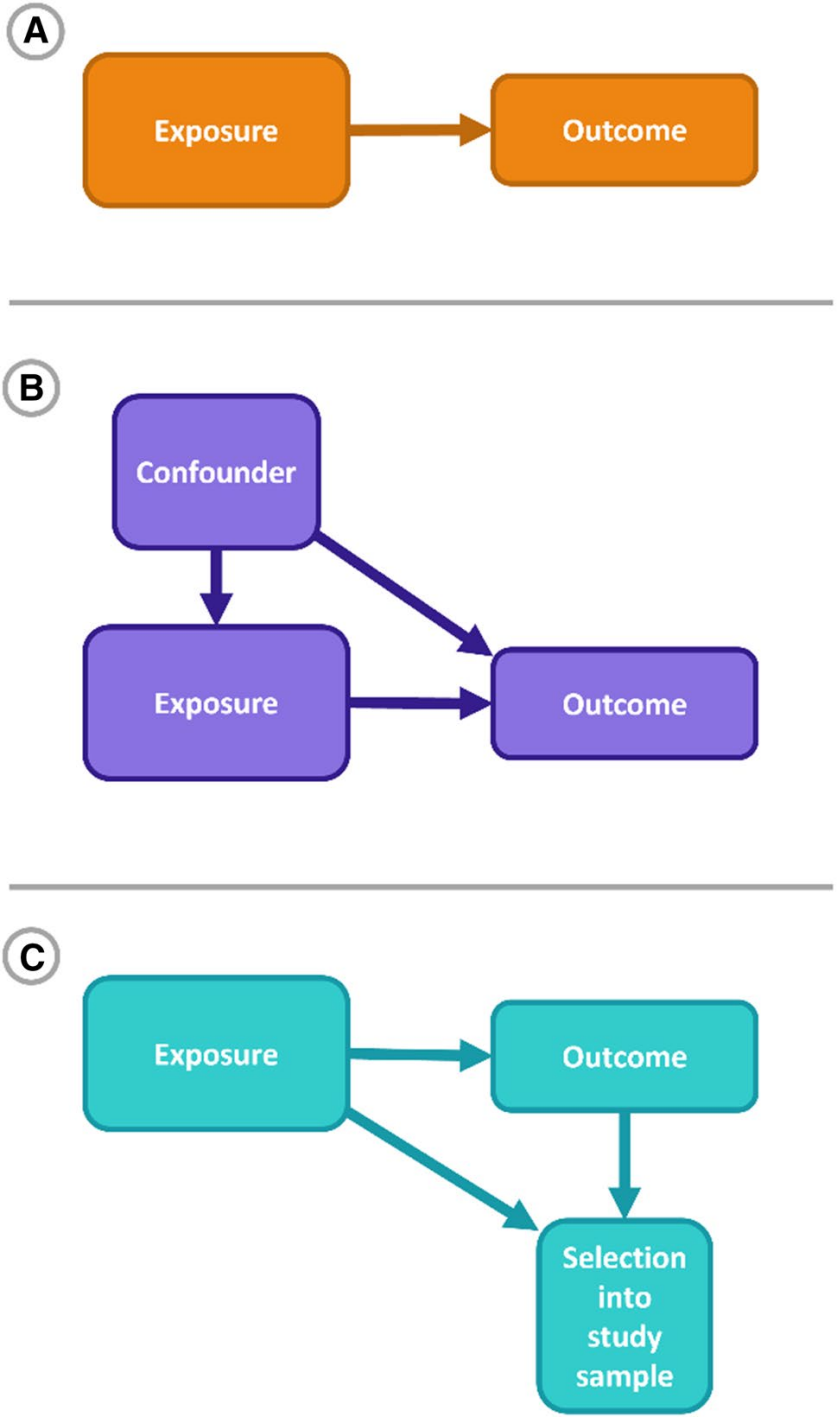
Illustrative causal diagrams (Directed Acyclic Graphs) for exposure-outcome associations in three studies testing the same association. Cross-study differences could be due to: **A** a causal effect of the exposure on outcome; **B** associations arising due to confounding by a third variable; and **C** an association biased due to sample selection

Differences in results across study populations could arise from multiple sources; care is therefore required in interpretation. Heterogeneity in the magnitude or direction of causal effect represent one potential explanation for between-study difference in association (Fig. 2A). Others include differences in (unobserved or residual) confounding between studies (Fig. 2B), or differences attributable to sample selection (Fig. 2C) [99]. For example, differences over time in the association between an exposure and an outcome could occur because: (1) the processes of selection into the exposure varies over time (e.g., the characteristics of individuals who engage in a certain behaviour change); or (2) the association between the exposure and the outcome is confounded by individual characteristics and the link between these individual characteristics and the outcome vary over time. Depending on the question of interest these alternative scenarios may be of substantive interest. For example, by facilitating investigation of age, period and cohort effects (APC) [21], cross-study research can also provide valuable insights on how macrosocial trends and demographic shifts shape social, economic and health outcomes. Period and cohort effects can be manifested in cross-study differences in association, but also in cross-study differences in selection to the exposure and/or confounding structure; each is of interest to better understand the processes which underlie social change.

Delineating between the different scenarios described above requires theoretical (ideally, evidence-based) understanding of the likelihood of each scenario, and where possible analytical strategies which test the robustness of the observed associations to confounding and differences in sample selection across studies (e.g., via statistical adjustment (and sensitivity analysis to note importance of residual confounding [100]), use of fixed effects analysis [101], negative controls [102], instrumental variable analysis [103], and/or use of genetically informed designs to aid inference [104]). Since causal inference in observational data relies on the plausibility of the assumptions required for each method used, multiple methods can be used with different assumptions and results compared—termed ‘triangulation’ [105] in epidemiology or comparison of ‘evidence factors’ in social science [106]. Even when the same magnitude of association is found in different studies, this may still reflect differences in the underlying processes in each study; for instance, a causal effect in one study, yet a confounded effect in another. Thus, care is also required to account for such causes of bias even where no cross-study differences are reported (null findings may be a product of such biases).

Causal effects may be context specific, particularly where they reflect or are dependent upon societal influences [107, 108]. For instance, associations between socioeconomic factors and outcomes such as body weight and smoking appear to have reversed in magnitudes across time in some high-income countries [25, 109], potentially reflecting differences in the direction of the causal effect between socioeconomic factors and outcomes. Even in contemporaneous populations in which strong social gradients in health are observed, the magnitude in the socioeconomic-health association may differ due to changes in the prevalence of factors which modify the strength of the effect. For instance, policy changes which disproportionately affect disadvantaged populations may either weaken or strengthen the influence of socioeconomic factors on health. Broader societal changes may also lead to changes in the magnitude of inequalities observed; educational attainment may be a less distinguishing predictor of health in societies in which large fractions of the population are university educated, and may further depend on the (historically sizable) economic benefits of higher education [110].

In summary, cross-study differences may arise through multiple processes; when interpreting results, and considering their potential implications, these should be scrutinised and acknowledged where applicable (see Table 1).

## Conclusion

Comparative cross-study research initiatives are an increasingly prominent component of the social and health research landscape, yet they present considerable practical, analytical and conceptual challenges. We sought to address this through a multi-strand approach, focused on investigation of change across time in prevalence and association: by: (i) offering structured discussion on the diverse obstacles and opportunities involved in such work; (ii) leveraging that structure as the basis for a new framework/checklist; and (iii) providing an online teaching resource comprising annotated guidance and reusable analytical materials for newcomers to such research endeavours. This framework can inform and guide on-going conversation and debate on best practice in this field. Given the continued need to understand how social and health phenomena change across time and increasing numbers of initiatives to harmonise and make available data from different studies across time and place [4, 8, 111, 112], we anticipate that such research will remain fruitful in future.

## Supplementary Information

Below is the link to the electronic supplementary material.

Supplementary file1 (DOCX 21 KB)
