# Supplementary material for: Investigating change across time in prevalence or association: the challenges of cross-study comparative research and possible solutions
Source: Discov Soc Sci Health. 2022 Oct 27;2(1):18. doi: 10.1007/s44155-022-00021-1 (PMC9613735; doi:10.1007/s44155-022-00021-1)
Supplement: Supplementary file 1 — Supplementary file1 (DOCX 21 KB) [file 44155_2022_21_MOESM1_ESM.docx]

**Supplementary Table 1. An example of a cross-study association examined in both relative (odds ratio) and absolute (risk difference) form**

|  |  | Year |  |  |  |
| --- | --- | --- | --- | --- | --- |
|  |  | 1990 |  | 2020 |  |
| Exposure |  | Unexposed | Exposed | Unexposed | Exposed |
| Number diseased | Yes | 100 | 150 | 120 | 164 |
|  | No | 100 | 50 | 80 | 36 |
| Total n |  | 200 | 200 | 200 | 200 |
| Odds disease |  | 1.00 | 3.00 | 1.50 | 4.56 |
| **Odds ratio** |  | **3.00** |  | **3.04** |  |
|  |  |  |  |  |  |
| Risk |  | 0.5 | 0.75 | 0.6 | 0.82 |
| **Risk difference** |  | **0.25** |  | **0.22** |  |

Note:

Statistical packages enable both to be estimated using concise inter-related commands, for instance:

|  | **Stata** | **R** |
| --- | --- | --- |
| **Relative measures of association** | logistic disease i.exposure | stats::lm(disease ~ exposure) |
| **Absolute measures of association; risk differences** | logistic disease i.exposure  margins, dydx(i.exposure) | x <- stats::lm(disease ~ exposure)  summary(margins::margins(x)) |
